# Preoperative predictors of postoperative complications after gastric cancer resection

**DOI:** 10.1007/s00595-019-01877-8

**Published:** 2019-09-18

**Authors:** Mitsuro Kanda

**Affiliations:** Department of Gastroenterological Surgery (Surgery II), Nagoya University Graduate School of Medicine, 65 Tsurumai-cho, Showa-ku, Nagoya, 466-8550 Japan

**Keywords:** Gastric cancer, Postoperative complication, Predictor, Gastrectomy

## Abstract

Risk management is becoming an increasingly important healthcare issue. Gastrectomy with lymphadenectomy is still the mainstay of treatment for localized gastric cancer, but it is sometimes associated with postoperative complications that compromise the patient’s quality of life, tolerability of adjuvant treatment, and prognosis. Parameters based exclusively on preoperative factors can identify patients most at risk of postoperative complications, whereby surgeons can provide the patient with precise informed consent information and optimal perioperative management. Ultimately, these predictive tools can also help minimize medical costs. In this context, many studies have identified factors that predict postoperative complications, including indicators based on body constitution, nutrition, inflammation, organ function and hypercoagulation. This review presents our current understanding and discusses some future perspectives of preoperatively identified factors predictive of complications after resection for gastric cancer.

## Introduction

Despite remarkable advances in surgical and anesthesiologic techniques, postoperative care, and interventional radiology associated with gastric cancer, gastrectomy still has a risk of serious postoperative complications, such as anastomotic leakage and intraabdominal abscess. These complications can impede recovery, delay the initiation of adjuvant chemotherapy, and compromise quality of life [1, 2]. Moreover, postoperative complications have been shown to adversely affect the overall and recurrence-free survival of patients after curative gastrectomy for gastric cancer; thus, some complications can be catastrophic for both short- and long-term outcomes [3–5]. The recently reported overall morbidity rates after resection for gastric cancer are 17.4–24.5% in East Asia and a slightly higher rate of 13.6–46% in Western countries [2, 6–9].

**Fig. 1:**
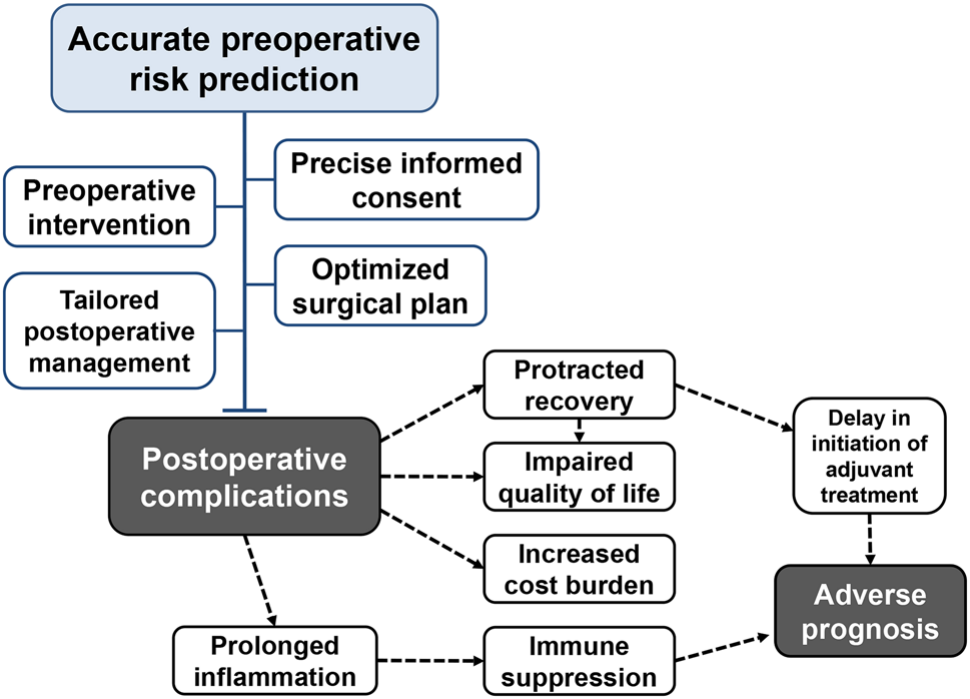
Importance of predictors for postoperative complications

Accurate risk stratification before surgery has the potential to improve several aspects of overall patient care, including more accurate informed consent, improved selection of procedures, better estimates of the likelihood of early and safe discharge, and more appropriate targeting of postoperative critical care services [10, 11] (Fig. 1). Therefore, it is crucial to design reliable and simple tools to predict postoperative complications. Several studies have identified an association between the incidence of postoperative complications and factors such as the postoperative elevation of inflammatory parameters [8, 12–14]. However, in most countries, surgeons are legally obliged to inform patients of the potential risks of surgery, which reduces the value of postoperative measures [15, 16]. The identification of patients most at risk of serious postoperative complications is essential to the decision-making process before surgery, highlighting the need to develop prediction tools based exclusively on factors identified preoperatively. Ideally, such tools should be based on simple, low-cost, rapid, and objective measures, and be applicable to all patients and hospitals.

Our aim is to provide an up-to-date review of some available predictive tools for postoperative complications after resection for gastric cancer, focusing on factors identified preoperatively. To this end, we reviewed publicly available literature sources and prioritized candidate predictive factors for discussion based on their simplicity, objectivity, and preoperative availability. This is a simple literature review that is neither a systematic review nor a meta-analysis. The articles selected met the following criteria, as described in the text: they focused on factors identified preoperatively to predict postoperative complications of gastric cancer resection; they evaluated predictive values in cohorts of 100 or more patients; and they were published from 2009 onward. The 14 candidate predictors are grouped into the following six categories: indicators of body constitution, nutrition, inflammation, organ function, hypercoagulation, and integrative risk models (Table 1).

**Table 1:** Preoperatively identified predictors of postoperative complications after gastric cancer resection

Category	Parameter	Measurement	Patients	Sample size	Type of relevant complications	Refs.
Body constitution	Body mass index	Body weight Height	Any stage	1249	Wound complications Non-abdominal complications	[20]
	Amount of visceral fat	Visceral fat area	pStage I/II/III	152	Overall complications	[17]
	Sarcopenia	Total abdominal muscle area Muscle strength	cStage I/II/III	470	Overall complications	[25]
Nutrition	Prognostic nutrition index	Total lymphocyte count Albumin	pStage I/II/III	7781	Overall complications	[41]
	CONUT score	Total lymphocyte count Albumin Cholesterol	pStage II/III	626	Non-abdominal complications	[46]
Inflammation	Systemic inflammation score	Lymphocyte–monocyte ratio Albumin	pT2–4	187	Overall complications	[51]
	Platelet–lymphocyte ratio	Platelet count Total lymphocyte count	cT2–4	312	Intraabdominal complications	[53]
	Neutrophil-to-lymphocyte ratio	Total lymphocyte count Neutrophil count	pStage I/II/III	404	Infectious complications	[56]
Organ function	eGFR	Creatinine Age	cT2–4	315	Anastomotic leakage	[59]
	Lung spirometry test	Forced expiratory volume Forced vital capacity	Any stage	538	Anastomotic leakage Wound infection	[62]
Hypercoagulation	Coagulation score	Fibrinogen D-dimer	pStage II/III	126	Intraabdominal complications	[65]
Integrative risk model	POSSUM	12 physiological variables 6 operative variables	Any stage	612	Overall complications	[70]
	SURPAS	8 physiological variables 4 operative variables 4 patient-related factors	Any stage Various diseases	1006	Overall complications	[75]
	Japanese National Clinical Database risk model	Age Sex Activity of daily living Body mass index Cardiovascular disease Albumin	Any stage Distal gastrectomy	65,906	Morbidities closely associated with mortality	[77]

*CONUT* Controlling Nutritional Status, *eGFR* estimated glomerular filtration rate, *POSSUM* Psychological and Operative Severity Score for the Enumeration of Mortality and Morbidity, *SURPAS* Surgical Risk Preoperative Assessment System

## Body constitution

Surgeons often assume that overweight patients are more likely than others to suffer short-term adverse events after gastric surgery [17]. Previous studies have discussed the influence of obesity on operative morbidity after gastric surgery and the feasibility that excess visceral fat increases the technical challenges of surgery, particularly during lymph node dissection [18, 19]. Body mass index (BMI) is one of the simplest indicators of physical constitution. Chen et al. evaluated the morbidity and mortality risks of 1249 gastric cancer patients undergoing gastrectomy based on the preoperative BMI (low < 18.5, normal 18.5–24.99, and high ≥ 25) [20]. They found that the overall postoperative morbidity risk was significantly higher for the high-BMI group (24.7%) and the low-BMI group (20.9%) than for the normal-BMI group (15.5%). Patients with a high BMI were more likely to have wound infections, abdominal hemorrhages, and cardiac complications, whereas those with low BMI had higher rates of mechanical obstruction, sepsis, pneumonia, and pleural effusion [20]. Kunisaki et al. used cross-sectional computed tomography at the level of the umbilicus to measure preoperative subcutaneous and visceral fat areas in 152 patients who underwent laparoscopy-assisted distal gastrectomy for early gastric cancer [17]. They found that a high visceral fat area was an independent predictor of conversion to open surgery as well as postoperative complications [17].

There is increasing evidence that another physical factor: sarcopenia, increases the risk of adverse postoperative outcomes for patients undergoing gastric surgery [21, 22]. Sarcopenia is characterized by loss of skeletal muscle mass and strength and is a major contributor to overall frailty [23, 24]. Huang et al. conducted a prospective study of patients who underwent radical gastrectomy for gastric cancer, to investigate the impact of preoperative sarcopenia on postoperative morbidity rates [25]. A cross-sectional computed tomography image at the inferior aspect of the third lumbar vertebra was evaluated to estimate total abdominal muscle area, handgrip strength was used as a measure of muscle strength, and the 6-meter usual gait speed was used as a measure of physical performance. Of the 470 patients included in the study, 20.6%, 10%, and 6.8% were identified as having pre-sarcopenia, sarcopenia, and severe sarcopenia, respectively. The authors of that study found that postoperative complications and duration of the hospital stay increased with advancing sarcopenia. Moreover, severe sarcopenia was an independent risk factor for all complications [25]. A prehabilitation program is recommended for patients with preoperative sarcopenia to enhance their functional capacity and improve their ability to withstand operative stress [24].

## Nutrition

Patients with gastric cancer are frequently malnourished [26]. In addition to the effects of poor oral nutritional intake and protein loss from the primary lesion, cancer cells secrete cytokines, such as tumor necrosis factor-alpha, which adversely affect catabolic metabolism [27–31]. Malnutrition renders patients more susceptible to infection, prolongs wound healing, and increases the risk of postoperative complications [32–35]. Thus, a proper preoperative assessment of the nutritional status of gastric cancer patients should be performed. Numerous studies have sought to develop a reliable, valid scoring system that can identify patients with poor nutritional status and some systems have been used successfully to predict complications after gastrectomy [36–38].

The prognostic nutrition index (PNI), which reflects a patient’s nutritional and immune status, is widely accepted for the prediction of surgical outcomes for patients with various solid organ cancers, including esophageal, colorectal, liver, and pancreatic cancer [39, 40]. Lee et al. conducted a large-scale retrospective analysis of 7781 patients who underwent gastrectomy for stage I–III gastric cancer to assess the value of PNI as a predictor of perioperative morbidity [41]. They found that a preoperative PNI of < 46.7 was an independent predictor of postoperative complications. Several studies have since reported consistent results, highlighting the value of PNI as a well-balanced indicator of nutrition and immunity [42, 43].

The Controlling Nutritional Status (CONUT) score provides a comprehensive evaluation of protein and lipid metabolism and immunocompetence, and is widely used to select patients for intervention by nutritional support teams and to predict patient response to nutritional therapy [44, 45]. Ryo et al. investigated the predictive value of the preoperative CONUT score in 626 patients with stage II/III gastric cancer and found that a score ≥ 2 was associated with a higher overall incidence of clinically relevant postoperative complications, particularly pneumonia [46]. PNI and CONUT scores are both based on mathematical equations, can be measured from single blood collections, are judged objectively, and are safely applicable in the clinical setting.

## Inflammation

Although preoperative systemic inflammation has been reported to predispose to postoperative infectious complications, there is limited evidence to support this [14]. Several studies indicate that *postoperative* changes in systemic inflammation markers, including C-reactive protein (CRP) and total white blood cell count (WBC), are useful to identify patients at risk of infectious complications [47–49]. In contrast, other studies have found that *preoperative* CRP, WBC, and the Glasgow Prognostic Score do not have significant predictive value for postoperative complications, whereas the preoperative systemic inflammation score (SIS), platelet–lymphocyte ratio (PLR), and neutrophil-to-lymphocyte ratio (NLR) do [32, 46].

SIS is based on the serum albumin level and the lymphocyte-to-monocyte ratio and is scored simply as 0, 1, and 2 [50]. Sato et al. investigated the relationship between preoperative SIS and postoperative complications in 187 previously untreated patients who underwent gastrectomy for pT2–4 gastric cancer and found a significant positive association between the SIS score and the incidence of complications [51]. One explanation for this is that a decreased lymphocyte count can compromise antimicrobial immune responses, contributing to increased infection with bacteria and other potential pathogens [50, 52].

Inaoka et al. retrospectively analyzed data from 312 patients with previously untreated clinical T2–4 gastric cancer who underwent a D2 standard gastrectomy [53]. They evaluated correlations between 21 candidate parameters measured by routine preoperative blood tests and clinically relevant postoperative complications and found that low preoperative PLR was highly specific/sensitive for predicting postoperative complications and was an independent risk factor in a multivariate binomial logistic analysis that included other potential risk factors. Of note, low preoperative PLR was significantly linked to the increased prevalence of intraabdominal complications, regardless of age, BMI, type of gastrectomy, or clinical disease stage. A low PLR is indicative of compromised cell-mediated immunity and malnutrition (reduced total lymphocyte count) as well as increased inflammation and thromphophilic diathesis (increased platelet count) [54].

NLR is a useful marker of subclinical inflammation [55]. Mohri et al. examined the association between postoperative complications and preoperative NLR in 404 patients undergoing curative resection for gastric cancer [56]. They found that a high preoperative NLR was independently predictive of the development of postoperative infectious, but not noninfectious complications. A high NLR is indicative of increased neutrophil-dependent inflammatory responses and decreased lymphocyte-mediated immunity, such as antibacterial responses [55]. Patients with various cancers, including gastric cancer, often have increased serum levels of the proinflammatory cytokine interleukin-6, which promotes the proliferation of immature neutrophils and stimulates mature neutrophils to release superoxide anions as a reaction to surgical trauma [57, 58]. Thus, high neutrophil levels may contribute to oxygen radical-mediated tissue injury and bacterial invasion postoperatively [56].

## Organ function

Renal function is routinely evaluated preoperatively to determine the optimal dose and infusion rates of medications. Tanaka et al. retrospectively analyzed the preoperative estimated glomerular filtration rate (eGFR) in 315 previously untreated patients who underwent curative D2 gastrectomy for clinical T2–T4 gastric cancer [59]. They found that a preoperative eGFR < 63.2 ml/min/1.73 m^2^ was an independent risk factor for postoperative complications. Specifically, anastomotic leakage was higher in patients with a low eGFR than in those with a high eGFR (9.4% vs. 3.5%). Low eGFR might reflect an overall decline in the function of major organs, which would compromise the patient’s ability to resist complications [60]. Even in patients with subtle renal disease, a deterioration in drug metabolism/excretion and edema of the tissues resulting from water–electrolyte imbalance may result in poor wound healing and infection control, ultimately leading to the development of serious complications [59, 61].

Lung spirometry is also routinely performed before major surgery to evaluate respiratory function. Jeong et al. analyzed the predictive value of preoperative spirometry results for postoperative morbidity in 538 gastric cancer patients who underwent gastrectomy [62]. They found that patients with abnormal pulmonary function (forced expiratory volume in 1 s/forced vital capacity of < 0.7) had a significantly higher incidence of local (29.9% vs. 18.1%) and systemic (8.2% vs. 2.0%) complications than patients with normal pulmonary function. Among the local complications, anastomosis leakage and wound complication were more common in patients with abnormal pulmonary functions. These findings suggest that preoperative spirometry may help identify patients with gastric cancer who are candidates for respiratory rehabilitation, as well as provide patients and their treating professionals with decision-making information regarding the potential benefits of surgery [63].

## Hypercoagulation

Hypercoagulation induces the formation of microthrombi, leading to tissue ischemia, impaired wound healing, and an increased risk of severe complications [64]. We previously designed a coagulation score based on fibrinogen and D-dimer levels (0: both below upper limits; 1: either above the upper limit; 2: both above the upper limits) and evaluated its predictive value for complications after curative gastrectomy in patients with stage II/III gastric cancer [65]. We found a positive relationship between the prevalence of postoperative complications and the coagulation score, with complication rates of 8%, 20%, and 27% for patients with scores of 0, 1, and 2, respectively.

## Integrative risk models

Traditional methods of identifying postoperative complications have relied on voluntary reporting by patients or incident reporting by physicians, but these approaches have often failed to detect and/or correctly identify complications [66, 67]. Various risk scoring systems have been introduced to identify surgical complications, including the American Society of Anesthesiologists (ASA) classification, APACHE (Acute Physiology and Chronic Health Evaluation), and POSSUM (Psychological and Operative Severity Score for the Enumeration of Mortality and Morbidity) [66, 68, 69]. Hong et al. compared the performance of POSSUM and APACHE II scoring systems for predicting surgical morbidity in 612 gastric cancer patients undergoing gastrectomy and found that the rate predicted by POSSUM, 36.6%, was close to the actual rate of 34% [70]. However, these scoring systems have limitations, such as interobserver variation (ASA), complexity (APACHE), and overestimation of mortality in lower risk groups (POSSUM). Subsequently, several integrative risk models have been developed [66, 67, 71].

The Surgical Risk Preoperative Assessment System (SURPAS) clinical decision support system was developed from the American College of Surgeons National Surgical Quality Improvement Program dataset [72]. SURPAS provides an individualized preoperative risk assessment for 30-day postoperative adverse outcomes in eight areas: (1) mortality (2) overall morbidity, and six complication clusters, namely (3) infectious (4) transfusion and cardiac (5) renal (6) pulmonary (7) venous thromboembolic, and (8) neurological complications [72]. The risk assessments are based on eight preoperatively available predictor variables, four of which are operative characteristics and four of which are patient characteristics, including ASA class and age [73]. On completion of the data input, the tool automatically displays the patient’s individual calculated risk for each postoperative outcome compared with the average patient undergoing the same operation [72, 74]. Khaneki et al. evaluated the performance of the SURPUS in 1006 patients and demonstrated more accurate risk predictions than revealed by the other risk model: the American College of Surgeons Surgical Risk Calculator [75]. However, SURPAS has some limitations when applied to gastric surgery, including lack of specificity to gastrectomy, interobserver variation in the ASA component, and a high burden of labor from the number of evaluation items. Large-scale validation of SURPAS in populations of different races and ethnicities is desirable before this tool is popularized.

In Japan, a risk model to predict 30-day mortality (death within 30 days after surgery, in hospital or outside) and operative mortality (death during up to 90 days of hospitalization) was constructed using data from the National Clinical Database: a nationwide web-based database of 33,917 patients who underwent distal gastrectomy in Japan in 2011 [2, 76]. One limitation of this model is that, although it is an automatic calculation system, only the risks of mortality are estimated. Employing the Japanese National Clinical Database model, Kunisaki et al. sought to identify preoperative risk factors that predict eight major postoperative complications associated with mortality, including unplanned intubation, pneumonia, systemic sepsis, renal failure, cardiac events, blood transfusions > 5 units, central nervous system events, and anastomotic leakage [77]. They analyzed data from 65,906 patients who underwent distal gastrectomy during 2011 and 2012 and identified a number of predictive risk factors that were subsequently reproduced and confirmed with a 2013 validation dataset [77]. Despite the large number of patients included, the clinical value of these predictors is unclear, because each complication was evaluated separately.

## Clinical implications and future perspectives

Despite extensive investigation of several preoperative parameters for their ability to predict postoperative morbidity rates, less is known about the predictive factors for postoperative complications. In this review, we discussed some promising predictors, selected based on their simplicity, low cost, amenability to rapid evaluation, objectivity, and preoperative availability. A comprehensive evaluation of patients using these predictors would undoubtedly improve the quality of perioperative management and encourage personalized care of gastric cancer patients.

The conventional integrative risk models, such as ASA classification, POSSUM, and APACHE II, arguably have greater predictive value than single parameters; however, they are complex, labor-intensive and time-consuming, and unlikely to be used routinely in daily clinical practice [66, 67, 78]. In contrast, single parameters have the advantage of simplicity, but the drawback of requiring optimized cutoff values. Because most analyses of predictors of postoperative complications have been retrospective with relatively small sample sizes, their reproducibility and universality have yet to be demonstrated. Large-scale prospective studies are needed to establish reliable cutoff values for single-parameter predictors.

Potentially, there are two ways for single parameters to be utilized clinically. First, tests measured in the preoperative workup can be taken into account in nomograms, alone or in combination with other simple parameters. This would lead to decreased false negative rates, facilitate the physician’s decision-making, and provide the patient with more complete informed consent before gastrectomy. Nevertheless, unless they are reduced, the time and labor costs of this approach would be comparable to those of conventional integrative risk models. Some of the key factors in establishing new integrative risk models are the inclusion of only objective and preoperatively measured parameters, and the development of automatic calculation systems connected to medical records. Second, single parameters could be used not only as variables for evaluation, but also as eligibility criteria for prospective clinical trials of the benefits of nutritional support, anti-coagulation agents, and anti-inflammation therapy for improving the safety of surgery.

Intense effort has been expended to identify factors related to adverse surgical outcomes, several of which, including medical comorbidity, old age, combined resection, and advanced disease stage, have been shown to be associated with postoperative complications. However, these factors are intrinsic to the patient’s physical or disease status and cannot be changed before surgery. Nevertheless, several factors have been identified that can be improved preoperatively, such as nutritional, inflammatory, and hypercoagulable status, physical condition, and organ function. Whether normalization of these factors can reduce postoperative complications is still under investigation. Compelling data support the notion that providing preoperative nutritional support can strengthen immunity, suppress postoperative inflammation, decrease complications, and enhance tolerance to adjuvant chemotherapy [41, 79, 80]. The value of preoperative modification of systemic inflammation and hypercoagulable conditions in reducing postoperative complications is currently unknown. Thus, prospective clinical trials of anti-inflammatory and anti-coagulation treatments are warranted.

An accurate preoperative estimation of the risks for complications can improve patient management through the modification of surgical plans. First, physicians can establish appropriate timing of surgery based on the necessity of preoperative interventions. Second, extensive surgical procedures, such as combined resection of the pancreas and extended lymphadenectomy, should be precluded for patients at risk. Third, wedge or segmental resection of the stomach should be considered as an alternative to distal or total gastrectomy for patients at a high risk for severe complications or mortality. Finally, treatment options other than surgery should be recommended for patients at critically high risk.

## Conclusion

**Fig. 2:**
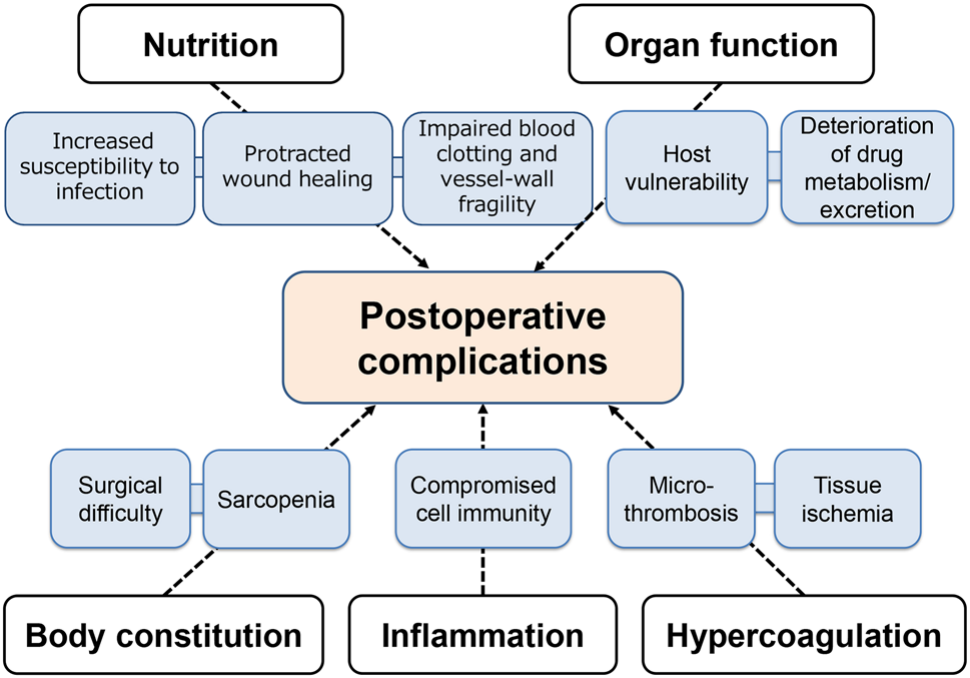
Potential contributors to the development of postoperative complications after gastrectomy

This review summarizes the association between several patient-related factors and postoperative complications in patients undergoing resection for gastric cancer and evidence suggests that interplay among these factors might increase the risk of complications further (Fig. 2). It is difficult to conclude which predictor is the most reliable tool, because direct comparisons among the candidate predictors have not been performed and the five categories have a crosstalk with each other and all contribute to the development of complications after gastrectomy. Further development of integrative systems employing the candidate predictive tools described in this review will enable the identification of patients at risk before surgery, thereby advancing the quality of perioperative management and overall clinical care, encouraging individually tailored care, and improving outcomes for patients with gastric cancer.
