# Correction to: Preoperative predictors of postoperative complications after gastric cancer resection

**DOI:** 10.1007/s00595-019-01948-w

**Published:** 2020-01-08

**Authors:** Mitsuro Kanda

**Affiliations:** Department of Gastroenterological Surgery (Surgery II), Nagoya University Graduate School of Medicine, 65 Tsurumai-cho, Showa-ku, Nagoya, 466-8550 Japan

## Correction to: Surgery Today (2020) 50:3–11 10.1007/s00595-019-01877-8

The article Preoperative predictors of postoperative complications after gastric cancer resection, written by Mitsuro Kanda, was originally published Online First without Open Access. After publication in volume 50, issue 1, page 3–11 the author decided to opt for Open Choice and to make the article an Open Access publication. Therefore, the copyright of the article has been changed to © The Author(s) 2020 and the article is forthwith distributed under the terms of the Creative Commons Attribution 4.0 International License (https://creativecommons.org/licenses/by/4.0/), which permits use, duplication, adaptation, distribution and reproduction in any medium or format, as long as you give appropriate credit to the original author(s) and the source, provide a link to the Creative Commons license, and indicate if changes were made.

The original article has been corrected.

